# News and Coming Events

**Published:** 1909-05-15

**Authors:** 


### News and Coming Events.

Mr. Ernest Mallam, D.M., Magdalen College, has been appointed Litchfield Clinical Lecturer in Medicine in the University of Oxford for a term of two years from October, 1909.

Lord Sandhurst, Treasurer of St. Bartholomew's Hospital, has received from the Merchant Taylors' Company £500, being the first moiety of a further grant of £1,000 towards the building fund of the hospital.

A loan exhibition of pictures by Jan Steen will be held at the Dowdeswell Galleries, 190 New Bond Street, London, W., next week, in aid of the National Hospital for the Paralysed and Epileptic, Queen Square, W.C.

The Brompton Hospital for Consumption has received from Washington cheques for one thousand dollars (£200) and five hundred dollars (£100), representing the prizes awarded to the hospital and the Frimley Sanatorium at the recent International Tuberculosis Congress.

The Council of the Senate of the University of Cambridge has reappointed Professor G. Sims Woodhead, M.D., as a Governor of Cheshunt College for three years, and as the representative of the University of Cambridge on the Council of the Lister Institute of Preventive Medicine.

Mr. Charles John Whittingham, of Chingford, who died on March 19 and left estate valued at £14,231 gross, bequeathed £250 to Guy's Hospital. He also left to this hospital the ultimate residue of his property, subject to the life interest of his wife and to other interests.

The fourth annual meeting of the Convalescent Homes Association will be held at 32 Sackville Street, W., on Thursday, May 20, at 4.30 p.m., Sir William S. Church, Bart., K.C.B., M.D., in the chair. The report for the year 1908 will be submitted, and the object and work of the Association will be explained.

The course of eight lectures on the "Structure and Functions of the Central Nervous System" given in the Physiological Institute of University College by Dr. W. Page May on Tuesdays at 5 p.m., which commenced on May 11, are open to all students of London University, as well as to medical men on presentation of their cards.

The Executive Committee of the British Institute of Social Service, 11 Southampton Row, W.C., has organised a Conference, to be opened by the Lord Mayor of London, on "Some Aspects of the Report on the Royal Commission on the Poor Laws and Relief of Distress," to be held in the Guildhall Council Chamber, by the kind consent of the Common Council, at 11.30 a.m. and 3 p.m. on Tuesday and Wednesday, May 18 and 19, 1909.

The annual general meeting of the Poor-law Medical Officers' Association of England and Wales will take place this year on July 6 at the Guildhall, London, and a conference will be opened there on the report of the Royal Commission on Poor-law Medical Relief. The Lord Mayor has promised to open this conference at 11 a.m. in the Council Chamber of the Guildhall. All Poor-law medical officers in England and Wales are invited to attend, and any desiring to read papers at the conference should send preliminary abstracts to the honorary secretary of the Association, Dr. M. Greenwood, 243 Hackney Road, London, N.E.

The eleventh meeting of the departmental committee appointed by the Lord President of the Council to consider the working of the Midwives Act was held at the Privy Council Office on May 5, Mr. Almeric W. FitzRoy, the Clerk of the Council, presiding. Evidence was tendered on behalf of the British Medical Association by the following witnesses:—Mr. J. Smith Whitaker, M.R.C.S., L.R.C.P., Medical Secretary of the Association; Mr. C. E. S. Flemming, M.R.C.S., L.R.C.P., Bradford-on-Avon; Dr. L. S. McManus, Wandsworth; and Mr. J. H. Taylor, M.B., Salford.

Under the will of the late Mr. E. Homan, of Lyme Regis, Dorset, St. Batholomew's Hospital will receive £1,000 towards the Samaritan Fund, and the Finchley Cottage Hospital will receive a like amount for endowment purposes, as well as £500 towards the Samaritan Fund. The late Mr. R. Haslam, of Burnley, has bequeathed to the Victoria Hospital, Burnley, £500 for the endowment of a cot. The late Miss A. E. Wheeler, of London, bequeathed £1,000 each to the National Hospital for the Paralysed and Epilepic and to the Royal Hospital for Incurables, Putney.

